# A Deep Learning Approach to Alzheimer’s Diagnosis Using EEG Data: Dual-Attention and Optuna-Optimized SVM

**DOI:** 10.3390/biomedicines13082017

**Published:** 2025-08-19

**Authors:** Funda Bulut Arikan, Dilber Cetintas, Aziz Aksoy, Muhammed Yildirim

**Affiliations:** 1 Department of Physiology, Faculty of Medicine, Kirikkale University, Kirikkale 71451, Turkey; 2 Department of Computer Engineering, Malatya Turgut Ozal University, Malatya 44210, Turkey; dilber.cetintas@ozal.edu.tr (D.C.); muhammed.yildirim@ozal.edu.tr (M.Y.); 3 Department of Bioengineering, Malatya Turgut Ozal University, Malatya 44210, Turkey; aziz.aksoy@ozal.edu.tr

**Keywords:** Alzheimer, artificial intelligence, deep learning, EEG, MobileNetV2

## Abstract

**Background/Objectives**: Alzheimer’s disease (AD) is a progressive neurodegenerative disorder, pathologically defined by the accumulation of amyloid-β plaques and tau-related neurofibrillary tangles in the brain. It represents a principal driver of cognitive deterioration in middle-aged and elderly populations. Early diagnosis and pharmacological management of the disease markedly improve both the quality and duration of life. **Methods**: Electroencephalography (EEG) is critical in detecting and analyzing Alzheimer’s disease. The widespread use of mobile EEG devices in recent years has necessitated real-time and effective data processing. However, extracting disease-specific features from EEG data still poses a significant challenge, especially in cases that must be completed quickly. This study aims to determine the frequency bands associated with Alzheimer’s disease in EEG data obtained from multiple channels and to accelerate the detection methods. An accurate classification that requires little computation is the primary goal. **Results**: EEG recordings of 48 individuals (24 AD and 24 healthy controls (HC)) obtained from Florida State University were divided into Alpha, Beta, Delta, Gamma, and Theta frequency bands; scalograms and spectrograms were generated for each frequency band. The effectiveness of these bands was evaluated using the MobileNetV2 architecture. The results showed that Delta and Beta frequency bands were the most significant for Alzheimer’s detection. By analyzing the features obtained from the Delta and Beta bands using the MobileNetV2 model integrated with the Dual-Attention Mechanism, it was determined that the attention mechanisms improved model performance by 2%. In addition, the use of an SVM classifier with hyperparameters optimized via Optuna resulted in approximately 3% performance improvement, suggesting that hyperparameter tuning may contribute positively to classification accuracy. Furthermore, combining features obtained from these frequency bands increased the detection performance when evaluated with larger datasets. **Conclusions**: The study demonstrates the potential of frequency band-based analyses and feature fusion methods to increase the accuracy and efficiency of Alzheimer’s diagnosis using EEG data. The results are promising; however, they should be interpreted with caution regarding their generalizability.

## 1. Introduction

Alzheimer’s disease, the most prevalent form of dementia worldwide, particularly in middle-aged and elderly populations, is a progressive neurodegenerative disorder characterized by two primary pathophysiological hallmarks: Positive lesions include the accumulation of amyloid-β plaques, tau-associated neurofibrillary tangles, dystrophic neurites, neuropil threads, and other abnormal protein aggregates. Negative lesions are marked by significant brain atrophy resulting from neuronal, neuropil, and synaptic loss [1,2]. Moreover, neuroinflammation, oxidative stress, apoptosis, and cholinergic neuronal damage have been proposed as key mechanisms potentially underlying the pathogenesis and progression of Alzheimer’s disease [3]. The predictable progression of Beta-amyloid (Aβ) plaque pathology from the neocortex, over limbic structures, diencephalon, and basal ganglia, to the brainstem and cerebellum reflects the spatiotemporal trajectory of disease advancement [4].

Although the pathophysiology of the disease is not yet clear, genetic predisposition and environmental factors are thought to play a role [3]. Alzheimer’s disease is characterized by memory impairment, progressive cognitive decline, and, in advanced stages, motor dysfunction, leading to increasing dependence in daily activities [4]. Clinical findings, imaging methods (MRI, PET, CT), and genetic tests are used to prevent this situation and provide early diagnosis. However, these methods are usually costly and time-consuming. The clinical diagnosis of Alzheimer’s disease typically involves multifaceted assessments conducted by specialist physicians. In this process, objective measurement tools such as blood tests, neurological examinations, and psychological tests for cognitive performance, brain imaging techniques including MRI and CT, and cerebrospinal fluid (CSF) biomarkers, as well as subjective elements such as the patient’s history and symptoms assessed through clinical observation, play important roles [1,5]. Interpreting these different diagnostic methods together requires expertise and a multidisciplinary approach. Early diagnosis of Alzheimer’s disease allows for the timely initiation of appropriate treatment approaches, which can slow disease progression and improve the individual’s quality of life [1,6]. In recent years, the analysis of EEG data has gained importance as a complementary biomarker in Alzheimer’s diagnosis due to its potential to detect cognitive impairment at an early stage.

EEG is a technique that allows the measurement of electrical activity in the brain, and a profile characterized by an increase in slow brain waves (Delta and Theta waves) is observed in Alzheimer’s patients [7]. EEG data from Alzheimer’s patients exhibit a significant slowing down, decreased synchronization, and low EEG signal strength compared to healthy individuals [8]. EEG stands out as a valuable tool for early AD detection with the potential to be integrated into multimodal diagnostic approaches [9]. EEG supports clinical decision-making processes by allowing early disease detection before physical symptoms appear. In addition, it does not involve radiation and is a cost-effective method, contributing to its wide application in the literature. Jain and colleagues evaluated the performance on a channel basis for AD detection by training EEG data from 19 electrodes with deep learning models such as SqueezeNet, ResNet, InceptionV3, and MobileNetV2. This study reported that the best results were obtained with the InceptionV3 model with 98.50% and 97.57% classification accuracy for P3 and T5 channels, respectively [10]. However, it was stated that considering EEG phases separately with this approach may negatively affect the holistic understanding of complex brain functions because analyzing EEG channels separately ignores the complex function and signal interaction in the brain.

Elgandelwar et al. compared traditional machine learning methods (SVM, DT, and KNN algorithm) with deep learning techniques (Multilayer Perceptrons [MLPs] and Convolutional Neural Networks [CNNs]). They showed that deep learning methods are more effective in diagnosing Alzheimer’s disease [11]. Imani et al. used ResNet, EEGNet, and DeepConvNet architectures to diagnose mild cognitive impairment (MCI), which is defined as the early stage of Alzheimer’s disease. Although 100% classification accuracy was achieved with the EEGNet and DeepConvNet models, the effectiveness of these models on different datasets was not tested. Imani and his colleagues used five selected EEG channels and multiplexing operations using the fusion of LSTM and CNN models and achieved a 98% accuracy rate [12]. In addition, the EEGConvNeXt model was proposed for the distinction of AD and Frontotemporal Dementia (FD), and this model achieved an accuracy rate of 95.70%. The architecture in question achieved success exceeding 98% in binary classification groups [13]. Kachare et al. employed a CNN architecture with a Softmax classifier on the Spanish EEG dataset comprising 12 AD patients and 11 healthy controls, achieving an accuracy of 98.05% [14]. Abadal et al. utilized Graph Convolutional Networks (GCNs) on a dataset including 63 individuals with Alzheimer’s disease, 63 with MCI, and 63 healthy controls, reporting an accuracy of 91.77% [15]. Zhou et al. proposed a Spatiotemporal Convolutional Gated Recurrent Unit (GRU) Network and validated their model using 10-fold cross-validation on a publicly available raw EEG dataset, achieving a notably high accuracy of 99.89% [16]. Similarly, Rostamikia et al. combined time and frequency domain feature extraction with Smirnov test-based statistical analysis and an SVM classifier on EEG recordings from AHEPA University Hospital, which included 29 healthy controls, 23 individuals with Frontotemporal Dementia (FTD), and 36 with Alzheimer’s disease, resulting in an accuracy of 93.5% [17]. Tara et al. present an EEG-based deep learning framework for classifying neurocognitive disorders (HC, MCI, AD, and Epilepsy) under eyes-closed conditions using PAC images and four CNN architectures. Sixteen key EEG channels were selected using Fisher’s score, and phase-amplitude coupling (PAC) was employed to capture cross-frequency interactions. Among the evaluated models, MobileNetV2 achieved the highest performance with an accuracy of 98.25% [18]. Another study proposes a lightweight and efficient CNN model for classifying cognitive states from EEG recordings. The approach involves a two-stage pipeline: first, generating 2D spectral images from EEG signals to capture spatial and spectral features; second, designing a compact CNN architecture using standard, depth-wise, and separable convolutions to optimize performance while minimizing computational cost. Evaluated on an open-access EEG meditation dataset, the proposed model achieves performance comparable to state-of-the-art methods while using less than 4% of their parameters, making it suitable for real-time applications like neurofeedback [19].

When the literature is examined, it is seen that the studies generally focus on efficient feature extraction from existing data due to the limited amount of data. However, the multi-channel structure of EEG data and the interactions of brain signals between channels still make the feature extraction process a significant challenge. In addition, it is noteworthy that existing studies largely focus on multiple or binary classifications between different neurodegenerative diseases (AD, FD, MCI). This study proposes a hybrid model using frequency bands that effectively detects Alzheimer’s disease without applying any multiplexing step. The approach in question aims to optimize the feature extraction and classification processes from EEG data and provide both limited data usage and more effective evaluation of interactions between channels. The main contributions of the study are as follows:

- With the widespread use of mobile EEG devices, an optimized model was developed to lighten the computational load and work quickly. The developed model contributed to the advancement of studies in this field by providing a basis for real-time EEG analysis.
- By using the Dual-Attention Mechanism, only the relevant features were evaluated, and the model aimed to learn information more efficiently with the attention mechanism.
- It has been analyzed with Continuous Wavelet Transform (CWT) and spectrogram methods, enabling the characteristic features in the signals to be revealed more comprehensively.
- Thanks to the hyperparameter optimization performed with Optuna, problems such as over-learning and inefficiency in the classification process were prevented, and significant improvements were achieved in model performance.
- In the model designed with a gradual structure, effective frequency bands were determined in the first stage, and these data were trained with a lightweight model.

This study aims to identify the frequency bands that are decisive in the diagnosis of Alzheimer’s disease and to evaluate the effectiveness of these frequency bands in classification processes. The study aims to reduce the cost of classification processes and obtain faster results by using only the determined effective frequency bands. This approach promises to both increase the efficiency of diagnostic processes and contribute to practical applications by reducing the computational load. In the continuation of the article, Section 2 and Section 3 are presented, and the article is concluded with Section 4.

## 2. Materials and Methods

### 2.1. Dataset

An EEG dataset created by Florida State University researchers and open to public access forms the basis of this study. EEG signals were obtained using a Biologic Systems Brain Atlas III Plus workstation with a 19-electrode recording setup in accordance with the international 10–20 system. The dataset includes 24 individuals diagnosed with Alzheimer’s disease and 24 healthy controls [19]. Recordings were performed in two different conditions, eyes open and eyes closed. The open eyes conditions are represented by groups A and C. The eyes-closed conditions are represented by groups B and D. Healthy individuals are included in groups A and B. In contrast, groups C and D consist of individuals diagnosed with Alzheimer’s disease according to the criteria of the “National Institute of Neurological and Communicative Disorders and Stroke (NINCDS-ADRDA)” and the “Diagnostic and Statistical Manual of Mental Disorders (DSM)” [20]. The dataset comprises EEG segments with a duration of 8 s and a sampling frequency of 128 Hz. All segments were preprocessed to remove ocular (e.g., eye blinks), motion-related, and myogenic artifacts, ensuring the integrity and reliability of the neural signals. A detailed analysis of the dataset, including demographic and experimental conditions, is presented in Table 1.

As detailed in Table 1, these groups were combined based on clinical diagnosis, irrespective of eye condition. This strategy was adopted to increase the sample size within each diagnostic category and to facilitate the development of a more robust and generalizable classification model. Although eye condition may introduce additional variability, we consider this variability as part of the inherent noise present in EEG data collected under clinical settings.

### 2.2. Method

**Figure 1:**
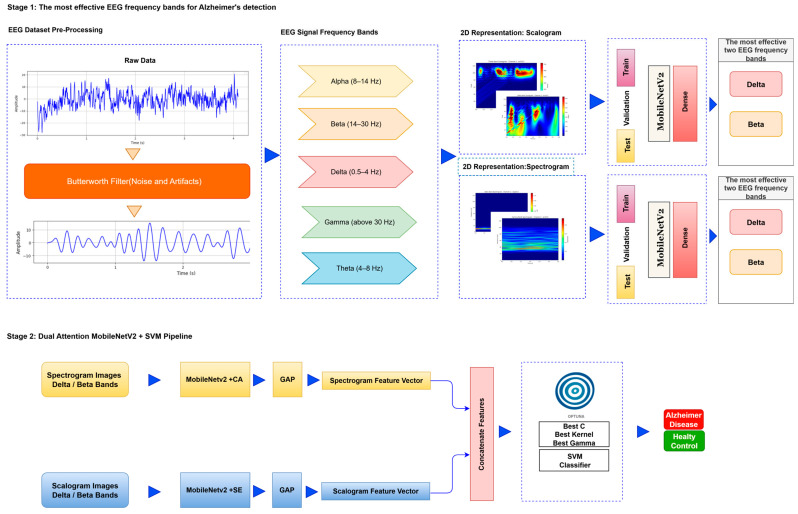
Proposed method.

The deep learning approach proposed for image-based analysis of Alzheimer’s disease consists of a series of systematic stages. First, preprocessing operations such as denoising and artifact cleaning are applied to the EEG signals to transform the raw data into a form suitable for analysis. This stage is followed by transforming the signals. Signals are transformed into time–frequency representations that reveal the dynamic properties of the signals by combining the time and frequency dimensions. This transformation is carried out by methods such as FT and CWT. The obtained time–frequency representations are divided into training, validation, and test data sets for the purpose of training, validation, and testing the deep learning model. Finally, these data sets are provided as input to a lightweight deep learning model. In the first stage, the most relevant EEG frequency bands for Alzheimer’s diagnosis are identified, allowing the analysis to focus solely on the most informative components rather than processing the entire dataset. In the second stage, features are extracted from these selected bands using a lightweight, frozen neural network integrated with attention mechanisms, and subsequently classified using an optimized SVM. The flow chart of the proposed model is presented in Figure 1.

Step 1: Preprocessing: EEG recordings are prone to artifacts such as eye movements, muscle activity, and network-related noise [21]. This study applied a 4th-order band-pass Butterworth filter (0.5–45 Hz) to remove the noise caused by direct current (DC) drift from the EEG signals and to obtain the relevant sub-bands. With this method, unwanted high-frequency noise was effectively eliminated.

**Figure 2:**
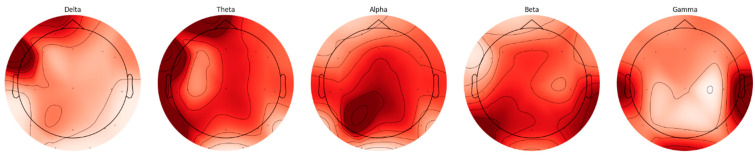
AD frequency bands topographic map.

Step 2: Segmentation to Frequency Bands: Signals were separated into frequency bands using wavelet-based algorithms to observe the changes in EEG sub-bands in Alzheimer’s disease and to determine which frequency band is effective in early diagnosis. To perform a more detailed analysis, the EEG signal of each channel was divided into specific frequency bands. Figure 2 shows the spectral distribution of frequency bands obtained from a relevant channel.

**Figure 3:**
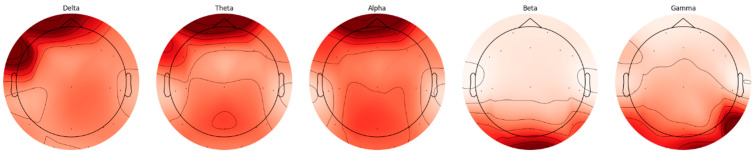
HC frequency bands topographic map.

EEG signals are divided into five basic frequency bands: Delta, Theta, Alpha, Beta, and Gamma [22]. The Delta band covers frequencies below 4 Hz, representing deep sleep and slow wave activities. The Theta band is in the range of 4–8 Hz and is associated with light sleep and relaxation states. The Alpha band reflects states of wakefulness, relaxation, and concentration in the range of 8–14 Hz. The Beta band is associated with active thinking, cognitive processes, and motor activities in the range of 14–30 Hz, while the Gamma band covers frequencies above 30 Hz and represents higher cognitive processes. The distributions of frequency bands on the scalp were evaluated separately for AD and HC, and topographic maps were created using the Welch method. The Weltch method calculates each channel’s power spectral density (PSD) in the specified frequency band. It draws a topographic map using each frequency band’s average PSD values and channel positions. These maps visualize the activity intensities of different frequency bands in the brain and are presented in Figure 2 and Figure 3.

When topographic maps are examined, a significant increase in low-frequency bands (Delta and Theta) is observed in individuals with AD. In contrast, a significant decrease in high-frequency bands (Beta and Gamma) is noted. In contrast, a more homogeneous distribution between frequency bands in HC individuals is exhibited.

Step 3: Time–Frequency Representation: In the proposed method, the model is evaluated using both scalogram and spectrogram images. Scalograms and spectrograms are created to include time and frequency domain information from EEG data. Scalograms are obtained using the wavelet transform. Wavelet transform provides an optimal time–frequency representation for non-stationary signals by using a wide window size for low frequencies and a narrow window size for high frequencies [23]. Scalograms of EEG data are created using the Morlet wavelet, which is known for its ability to effectively capture time–frequency features.

The spectrogram is a visual representation of the power density of a signal over time and allows the examination of the signal’s frequency components over time [24]. The Fourier Transform is used as the basis for creating spectrograms. This study analyzed the signal by dividing it into short intervals to apply the Fourier Transform. Each period was subjected to windowing to minimize losses and ensure continuity, and then overlapping was provided between the windows to increase the accuracy of the analysis process. The frequency spectrum of each window was calculated, and the obtained spectral density values were visualized in decibels and presented as a spectrum map in the time–frequency plane.

After the creation of image representations, a total of (19 × 24 = 456 AD − 19 × 24 = 456 HC) images were obtained for each frequency band group (Alpha, Beta, Delta, Gamma, Theta) from 19-channel EEG data of 24 individuals in the AD and HC groups. These data were fed to the model by separating them into 80% training, 10% validation, and 10% testing for each frequency band group. The same data partitioning strategy was consistently applied to both the MobileNetV2-based feature extraction and the SVM classification stages. The dataset was divided into training, validation, and test subsets on a subject-independent basis, and this separation was strictly preserved throughout all stages of the pipeline. Consequently, there was no overlap between the training and test sets, thereby completely eliminating the risk of data leakage.

Step 4: Feature Extraction-Integrated Attention for MobileNetV2: The MobileNetV2 model has fewer parameters than other CNN architectures, making it more efficient to deploy on low-power devices [25]. The MobileNetV2 [26] transfer learning model was used for training and feature extraction. MobileNetV2 is a lightweight Convolutional Neural Network architecture specifically designed to operate with high efficiency on mobile devices and embedded systems with limited computational resources. This architecture employs depthwise separable convolutions, which decompose standard convolution operations into computationally less intensive sub-operations. As a result, it significantly reduces the number of parameters and computational cost without compromising classification accuracy [27]. Furthermore, MobileNetV2 incorporates inverted residual blocks with linear bottlenecks, enabling the efficient learning of complex feature representations [28]. Due to these architectural advantages, MobileNetV2 was selected in this study for the purpose of feature extraction. Moreover, effective model training in EEG signal analysis typically requires large-scale labeled datasets. However, the non-stationary and stochastic nature of EEG signals poses significant challenges to the development of such datasets. Additionally, inter-subject variability further limits the reusability and generalizability of trained models across different individuals. To mitigate these limitations, transfer learning has been employed, enabling knowledge acquired from one task to be transferred to a related task. This approach facilitates model adaptation with limited data while preserving robustness to individual differences [29]. In particular, the integration of transfer learning strategies within CNNs has proven beneficial for the diagnosis and classification of different stages of Alzheimer’s disease [30].

Alpha, Beta, Delta, Gamma, and Theta frequency bands were converted to scalogram and spectrogram images and classified one by one. As a result of ablation studies, it has been determined that Delta and Beta frequency bands play a decisive role in Alzheimer’s diagnosis. Accordingly, feature extraction was performed using MobileNetV2 models integrated with different attention mechanisms, with time and frequency domain images obtained from the Delta and Beta frequency bands. Since both spectrograms and scalograms represent time–frequency images, the feature extraction was performed using MobileNetV2 integrated with the SE attention mechanism for scalograms, and MobileNetV2 with Channel Attention Mechanism for spectrograms. The manner in which the representations (spectrograms vs. scalograms) are applied primarily depends on which dimension of information—frequency or time—is more dominantly and distinctively presented. Spectrograms, generated via the FT, typically emphasize frequency resolution. FT performs fixed-frequency analyses within time windows, facilitating the capture of inter-channel frequency patterns. Therefore, Channel Attention is well-suited for highlighting discriminative frequency-based information across different EEG channels.

In contrast, scalograms, derived from the CWT, capture more localized and fine-grained temporal variations through scale-adapted wavelets. This characteristic enables the extraction of time-sensitive discriminative features. Consequently, SE attention is more appropriate for emphasizing critical activations along the temporal dimension.

In recent years, attention mechanisms have been stated to be an effective method in increasing model performance in the field of computer vision [31]. The primary function of attention mechanisms is to emphasize meaningful information and suppress unimportant information. These mechanisms are generally implemented as spatial attention, Channel Attention, or a combination of both. In this study, SE [32] and CA were integrated into the MobileNetV2 model and used for feature extraction. The SE attention mechanism converts the input feature maps into a single vector via global average pooling in the compression step. Then it maps this vector to a smaller size via a fully connected layer [33].

The Channel Attention Mechanism aims to learn the importance of each channel in an input feature map and integrates this information into the model’s weighting. This process enables the model to learn the essential features and optimize its performance via the attention mechanism.

Step 5: Classification: In the final step, SVM is utilized for the classification process. The determination of hyperparameters in SVM is performed using Optuna [34]. Optuna participates in determining the best values by performing trial and error with different hyperparameter values. It provides better results than traditional methods such as Grid Search [29] or Random Search [35,36].

The Optuna framework is used to address the problems of parameter tuning difficulty, lack of self-learning ability, and the limited generalizability of models obtained with traditional methods when faced with multiple parameter inputs [37].

## 3. Experimental Results and Discussion

This study was carried out to reduce the computational cost and quickly reach high accuracy levels using effective frequency bands. Experimental studies were conducted on the Google Colab platform on a Tesla T4 GPU. Two different visualization methods, scalogram and spectrogram, were used for time–frequency representation. Input images were prepared with 224 × 224 pixel dimensions and three color channels and presented to the pre-trained MobileNetV2 architecture. Frequency bands were trained separately with the MobileNetV2 model and a hybrid approach. During the training and evaluation of the model, hyperparameters such as a categorical cross-entropy loss function, a batch size of 32, 30 epochs, and the Adam optimization algorithm were used. Model performance was evaluated comprehensively through metrics such as accuracy, sensitivity, Precision, and F1-Score, MCC [38]. Plots of training/validation accuracy and loss for the scalogram representations of EEG frequency bands, along with their confusion matrices, are presented in Table 2.

According to the values in Table 3, the Delta frequency band performance has 92% accuracy, 92% F1-Score, and 85% MCC values. The fact that the model made only one misclassification among AD cases demonstrates its high sensitivity and reliability. The Delta frequency band is followed by the Beta frequency band with 82% accuracy and balanced performance metrics. The shortest processing time of 966 s makes the Beta frequency band an effective alternative in cases where the balance between performance and processing speed is critical. While the Theta frequency band is considered an acceptable option under certain conditions, the Gamma and Alpha frequency bands are considered ineffective ranges for detecting this disease due to their low performance.

Plots of training/validation accuracy and loss for the spectrogram representations of EEG frequency bands, along with their confusion matrices, are presented in Table 4.

In Table 5, the Delta frequency band also showed superior performance with 97% accuracy, 97% F1-Score, and 95% MCC values in spectrogram-based analyses and provided the fastest results in terms of processing time. The second most successful results in spectrogram analyses were obtained with the Beta frequency band. Delta and Beta frequency bands stood out in spectrogram and scalogram-based analyses by providing reliable results. Spectrogram-based analysis is more advantageous than scalogram-based analysis, especially regarding processing time. In addition, while the Alpha frequency band showed higher performance with spectrogram, the Gamma and Theta frequency bands showed relatively lower performance in both methods. As a result of these evaluations, it is appropriate to use Delta and Beta frequency bands in disease detection with spectrogram-based analyses.

The hybrid model in Table 6 exhibited a balanced and firm performance in the AD and HC classes. However, the false negatives observed in the AD class, i.e., the classification of some Alzheimer’s patients as healthy, constitute a critical problem in terms of healthcare applications, and reducing this situation is an issue that needs to be emphasized to increase the reliability of the model. However, these results obtained with a limited dataset without any data augmentation process reveal the model’s potential and provide a promising basis for further studies. In this context, our future studies plan to reach higher accuracy rates by expanding the dataset and optimizing the model parameters.

The observed decline in classification accuracy when combining Delta and Beta bands primarily stems from architectural differences. While the Delta-only result was obtained using an end-to-end fine-tuned MobileNetV2, the hybrid model employs a frozen MobileNetV2 as a feature extractor, followed by an external SVM classifier. This design precludes end-to-end optimization, limiting the model’s capacity to adapt feature representations to the target classification task.

**Figure 4:**
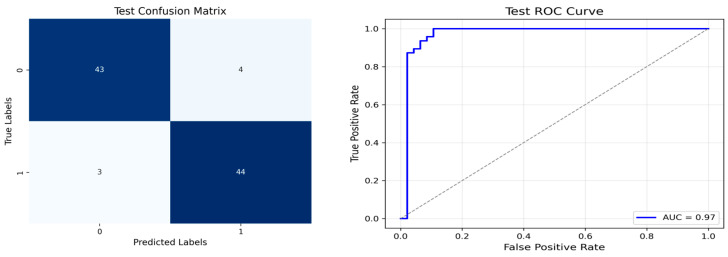
Confusion matrix and ROC for Delta + Beta combined features using MobileNetV2 and SVM.

The performance metrics in Table 6 demonstrate the effectiveness of combining the features obtained using Delta and Beta frequency bands with the MobileNetV2 model and the SVM classifier. This approach combined the strengths of both frequency bands to obtain robust classification results. The complexity matrix and ROC for Delta + Beta joint features using MobileNetV2 and SVM are presented in Figure 4.

**Figure 5:**
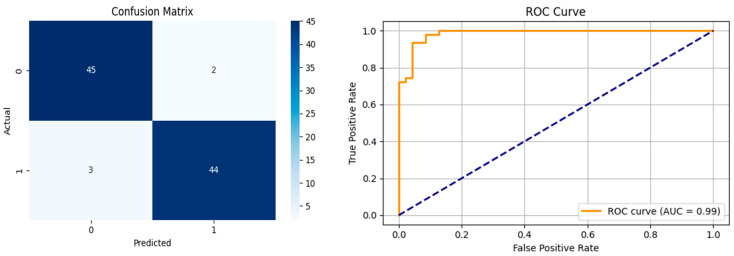
Confusion matrix and ROC analysis for combined Delta and Beta features using Integrated Attention Mechanism in MobileNetV2.

**Figure 6:**
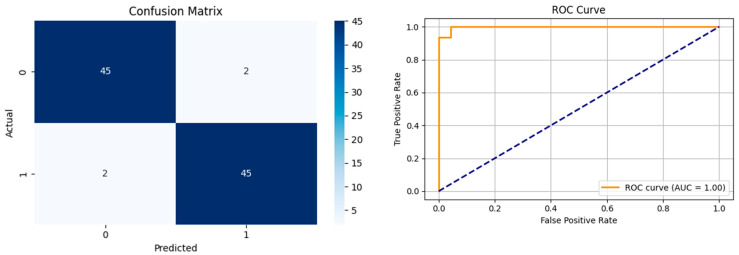
Confusion matrix and ROC analysis for combined Delta and Beta features using Integrated Attention Mechanism in MobileNetV2 and Optuna-optimized SVM.

Figure 5 shows that our model, equipped with an Integrated Attention Mechanism, achieves high performance in distinguishing between the two classes with minimal false predictions. The addition of Optuna-optimized SVM increased the Recall for the positive class by reducing the number of false negatives from 3 to 2. Figure 6 demonstrates the robustness of our Optuna-optimized model in maintaining accuracy while increasing Precision. The addition of an Optuna-optimized SVM significantly improved the Recall for the positive class and reduced the number of missed positive predictions (false negatives) by 33.3%. This improvement is especially valuable for applications where correctly identifying the positive class is critical, such as Alzheimer’s disease diagnosis.

Table 7 compares the Integrated Dual-Attention Mechanism and the Integrated Attention Mechanism with Optuna-optimized SVM classifier approaches in Alzheimer’s disease classification. Table 7 compares two configurations using the same feature extractor and classifier, but with and without feature fusion optimization. The obtained results show that both attention mechanisms and optimized machine learning classifiers can achieve high accuracy and stability when integrated with lightweight structures such as MobileNetV2. Furthermore, consistent F1-scores in the Integrated Attention Mechanism + Optuna-optimized SVM method reflect the reduced risk of overfitting, and the model can produce more reliable outputs.

However, the aim of this hybrid approach is not solely to maximize accuracy, but also to explore whether comparable performance can be achieved with reduced inference time and lower memory usage, as reflected in our quantitative performance table. The results of the 5 cross-validations are presented in Table 8 and the quantitative performance results are presented in Table 9.

The MobileNetV2 + Attention + Optuna SVM model demonstrates notable advantages over all other evaluated architectures. It maintains a balance between parametric efficiency and high classification accuracy, making it a computationally effective solution.

This study supports the existing literature by demonstrating that EEG-based brainwave frequency features—particularly those within the Beta and Delta bands—can serve as reliable biomarkers for the diagnosis of Alzheimer’s disease (AD). Beta activity, commonly associated with alertness, focused mental activity, attention, and cognitive processing, has been found to decrease significantly in AD patients, especially within the frontal and parietal regions. This reduction aligns with cognitive decline and memory impairment, and is thought to reflect disruptions in neurotransmitter systems, particularly cholinergic dysfunction [39]. Notably, reduced Beta power in these cortical areas has been linked to cognitive impairment and is detectable even in the early stages of the disease [40]. Delta waves, typically dominant during deep sleep [39], show increased power in awake AD patients, which has been associated with neurodegeneration and synaptic dysfunction, particularly in parietal and central cortical regions [40,41,42]. The Delta/Theta power ratio has also demonstrated high sensitivity and specificity in distinguishing AD from mild cognitive impairment (MCI) [40,41]. Moreover, an increase in Theta activity—particularly in posterior regions—has been correlated with cognitive slowing and memory deficits. Elevated Theta power may reflect disease severity and serve as a predictive marker for the progression from MCI to Alzheimer’s disease [39,41,42]. Gamma activity, essential for neuronal synchronization and cognitive integration, is typically reduced in AD patients, indicating impaired neural communication and integration processes. Experimental studies have further shown that 40 Hz Gamma stimulation can reduce amyloid plaque accumulation [43]. Furthermore, EEG spectral characteristics may offer clinical value in predicting treatment response. For instance, patients with low Beta activity may respond more favorably to cholinergic treatments [44].

The findings of this study are consistent with these prior observations and offer additional evidence using the same Florida State University dataset. A comparative summary of related studies employing this dataset is presented in Table 10.

A comparative summary of recent studies employing the Florida State University EEG dataset (comprising 24 Alzheimer’s disease (AD) and 24 healthy control (HC) subjects) is presented in Table 10. Sharma et al. [45] utilized a combination of Graph Fourier Transform (GFT) and Discrete Wavelet Transform (DWT), achieving an accuracy of 98.9%. Similarly, Göker [46] applied Welch spectral features and a BiLSTM classifier, reporting 98.8% accuracy. In another study, Thomas [47] used FIR filtering in conjunction with classical classifiers such as SVM, Naive Bayes, and XGBoost, attaining a maximum accuracy of 96%. While prior studies reported marginally higher accuracies, it is important to note that computational performance indicators such as model complexity, training time, and deployment feasibility were not explicitly discussed. Therefore, a complete comparison in terms of computational efficiency is not possible. Initially, our fine-tuned MobileNetV2 achieved 96% accuracy. However, with the unfreezing and retraining of the last 20 layers of the pre-trained network, the model’s performance improved, reaching 97% accuracy. This indicates that selective fine-tuning contributes positively to model generalization and task-specific learning.

## 4. Conclusions

This study investigated the effectiveness of frequency band-based EEG analysis for detecting Alzheimer’s disease (AD) using low-complexity deep learning models. By decomposing EEG signals into Delta, Theta, Alpha, Beta, and Gamma frequency bands and employing scalogram and spectrogram representations, we evaluated their individual and combined contributions using the MobileNetV2 architecture. The experimental results revealed that Delta, Theta, and Beta bands carry the most discriminative information for distinguishing AD patients from healthy controls. The findings support the hypothesis that targeted frequency band analysis can facilitate fast and accurate diagnosis of AD from EEG data, aligning well with the growing demand for portable and real-time diagnostic tools. In addition, the Dual-Attention Mechanism integrated into the model increased the classification accuracy to 95%. The integration of the Optuna-optimized SVM classifier further increased this rate to 96%, resulting in equal Precision, Recall, and F1-Score values for both AD and healthy individuals, thereby improving the balance. While the Optuna-optimized SVM classifier contributed to a marginal increase in performance, it is important to note that the improvement observed may be limited by the relatively small test set size. Therefore, further validation with larger and more diverse datasets is necessary to confirm the generalizability and statistical significance of these gains. Future work will focus on validating the proposed approach in more diverse clinical populations and optimizing the model for real-time deployment in wearable EEG systems. The proposed method offers a promising step toward developing efficient and accessible AD diagnostic support systems.

## Figures and Tables

**Table 1:** Dataset analysis.

Group	Condition	Number of Participants (n)	Mean Age	Age Range	Clinical Status	Eye Condition
A	Healthy Control	24	72	61–83	No neurological or psychiatric disorders	Eyes open
B	Healthy Control	24	72	61–83	No neurological or psychiatric disorders	Eyes closed
C	Alzheimer’s Disease	24	69	53–85	Diagnosed with probable AD (NINCDS–ADRDA and DSM-III-R criteria)	Eyes open
D	Alzheimer’s Disease	24	69	53–85	Diagnosed with probable AD (NINCDS–ADRDA and DSM-III-R criteria)	Eyes closed

**Table 2:** Plots of training/validation accuracy and loss for scalogram representations of EEG frequency bands, along with confusion matrices (predicted label and actual labels).

	Train/Validation Loss	ROC	Confusion Matrices
Alpha	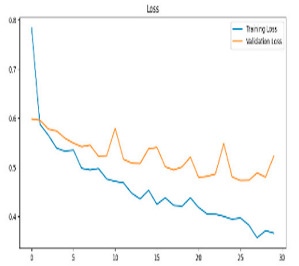	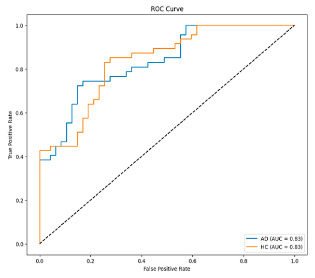	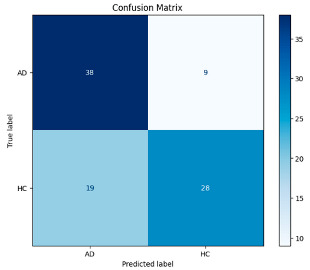
Beta	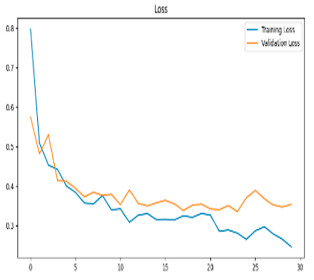	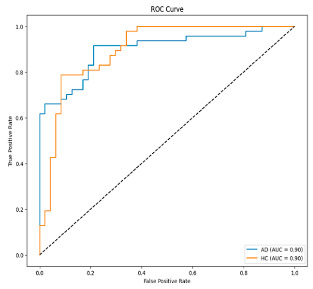	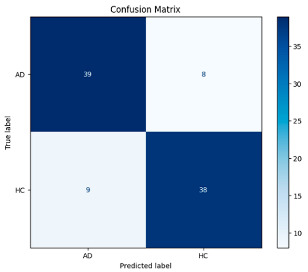
Delta	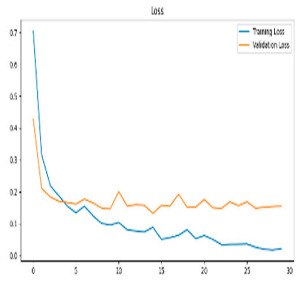	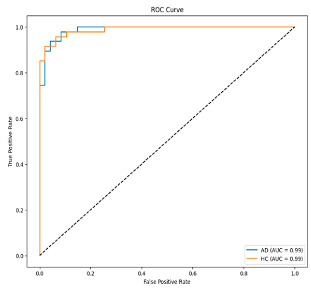	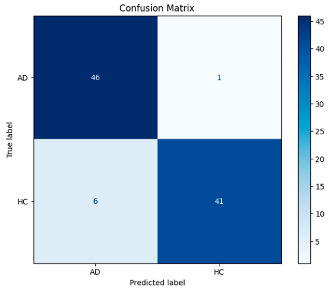
Gamma	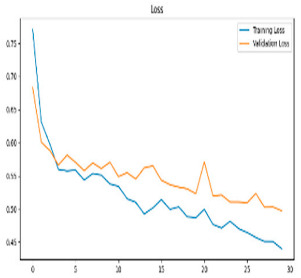	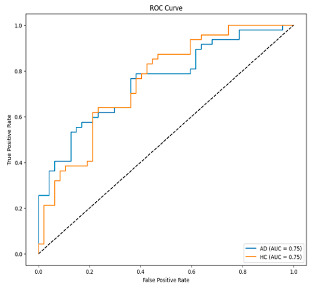	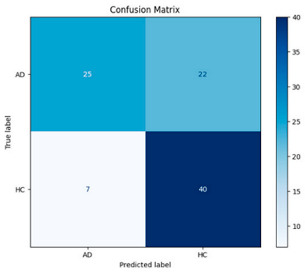
Theta	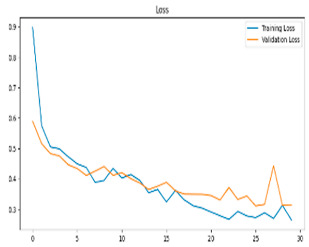	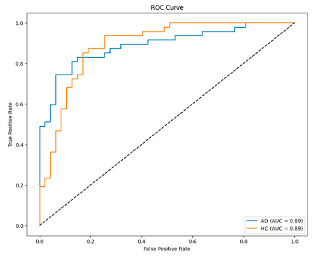	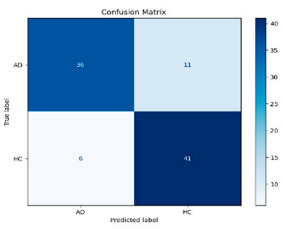

**Table 3:** Performance metrics for scalogram.

	Precision	Recall	F1-Score	Accuracy	MCC	Time(s)
Alpha	0.80	0.66	0.73	0.70	0.41	1144
Beta	0.82	0.81	0.82	0.82	0.63	966
Delta	0.97	0.88	0.92	0.92	0.85	1083
Gamma	0.53	0.78	0.63	0.69	0.40	1204
Theta	0.76	0.85	0.80	0.81	0.64	1152

**Table 4:** Plots of training/validation accuracy and loss for spectrogram representations of EEG frequency bands, along with confusion matrices (predicted label and actual labels).

	Train/Validation Loss	ROC	Confusion Matrices
Alpha	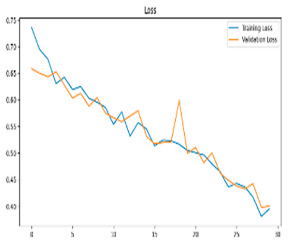	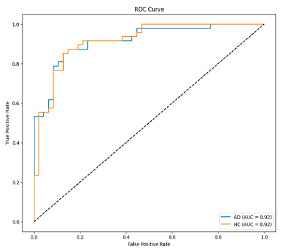	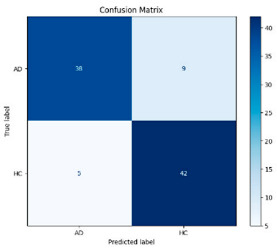
Beta	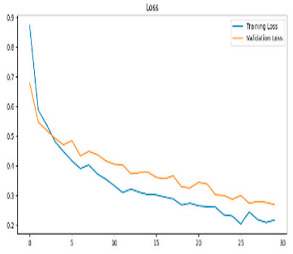	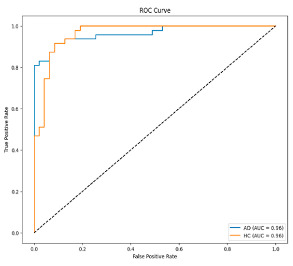	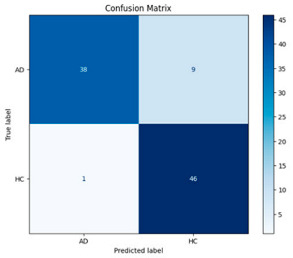
Delta	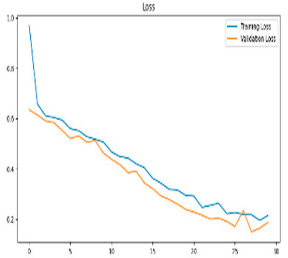	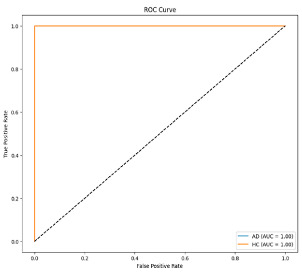	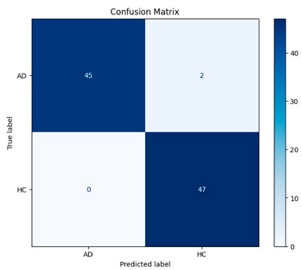
Gamma	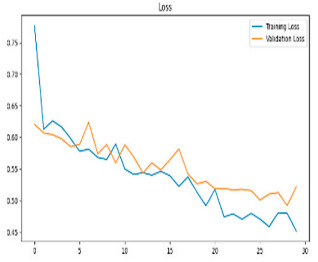	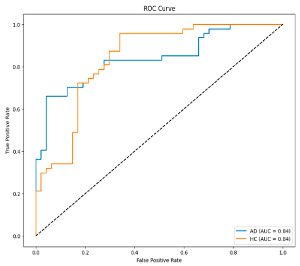	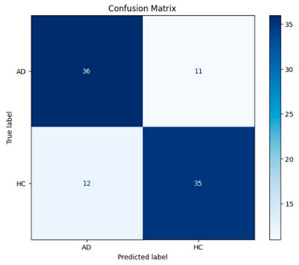
Theta	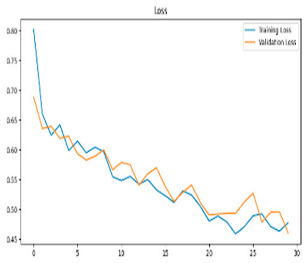	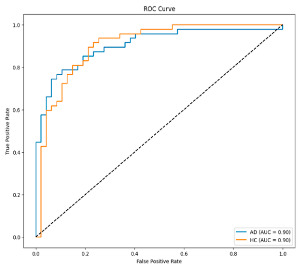	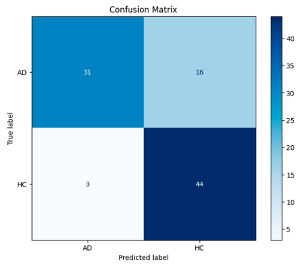

**Table 5:** Performance metrics for spectrogram.

	Precision	Recall	F1-Score	Accuracy	MCC	Time(s)
Alpha	0.80	0.88	0.84	0.85	0.70	637
Beta	0.80	0.97	0.88	0.89	0.79	615
Delta	0.95	1	0.97	0.97	0.95	611
Gamma	0.76	0.75	0.75	0.75	0.51	688
Theta	0.65	0.91	0.76	0.79	0.58	604

**Table 6:** Performance metrics for Delta + Beta combined features using MobileNetV2 and SVM.

	Precision	Recall	F1-Score	Accuracy
AD	0.93	0.91	0.92	0.93
HC	0.92	0.94	0.93	

**Table 7:** Comparison of performance with and without Optuna-optimized SVM.

**Integrated Dual-Attention Mechanism in MobileNetV2**				
	Precision	Recall	F1-Score	Accuracy
AD	0.94	0.96	0.95	0.95
HC	0.96	0.94	0.95	
**Integrated Attention Mechanism in MobileNetV2 and Optuna-Optimized SVM**				
	Precision	Recall	F1-Score	Accuracy
AD	0.96	0.98	0.97	0.97
HC	0.98	0.96	0.97	

**Table 8:** Results of 5 cross-validation.

	Precision	Recall	F1-Score	Accuracy	AUC
Fold1	0.98	0.96	0.97	0.97	0.99
Fold2	0.96	0.94	0.95	0.95	0.99
Fold3	0.94	0.92	0.93	0.93	0.98
Fold4	0.98	0.97	0.98	0.98	0.99
Fold5	0.96	0.95	0.96	0.96	0.99

**Table 9:** Quantitative performance metrics.

Model	Parameters	Model Size (MB)	FLOPS	Inference Time (ms) (One Image)	ACC
MobileNetv2+ Dense	2,422,210	10.85	-	4646.96	97%
Hybrid MobileNetV2 (Beta + Delta) + SVM	2,257,984	9.12	612,726,208	2798.45	93%
MobileNetV2 + Attention + Optuna SVM	2,469,024	10.70	613,672,288	3000.27	97%

**Table 10:** Comparison of results obtained from the same dataset.

References	Datasets	Methods	Results (Accuracy)
Sharma et al., 2024 [45]	Florida State University dataset (24 ad, 24 HC)	GFT + DWT	98.9%
Göker 2023 [46]	Florida State University dataset (24 ad, 24 HC)	welch spectral + BiLSTM	98.8%
Thomas [47]	Florida State University dataset (24 ad, 24 HC)	FIR Filter + SVM, NB, and XGBoost	96%
Ours	Florida State University dataset (24 ad, 24 HC)	CWT + Fine-Tune MobileNetV2 + Attention + Optuna	97%

## Data Availability

A public dataset was used in the study [17].

## Abbreviations

AD	Alzheimer’s Disease
CNN	Convolutional Neural Network
CT	Computed Tomography
CWT	Continuous Wavelet Transform
DC	Direct Current
EEG	Electroencephalography
FD	Frontotemporal Dementia
FT	Fourier Transform
FTD	Frontotemporal Dementia
GCN	Graph Convolutional Network
GRU	Gated Recurrent Unit
HC	Healthy Controls
KNN	K-Nearest Neighbor
LSTM	Long Short-Term Memory
MCI	Mild Cognitive Impairment
MCC	Matthews Correlation Coefficient
MLP	Multilayer Perceptron
MRI	Magnetic Resonance Imaging
NC	Normal Control
PET	Positron Emission Tomography
PSD	Power Spectral Density
SE	Squeeze and Excitation
STFT	Short-Time Fourier Transform
SVM	Support Vector Machine
